# Upregulation of UCP2 Expression Protects against LPS-Induced Oxidative Stress and Apoptosis in Cardiomyocytes

**DOI:** 10.1155/2019/2758262

**Published:** 2019-04-28

**Authors:** Jinda Huang, Wanwan Peng, Yijun Zheng, Hu Hao, Sitao Li, Yu Yao, Yue Ding, Junliang Zhang, Juanjuan Lyu, Qiyi Zeng

**Affiliations:** ^1^Department of Pediatrics, Zhujiang Hospital, Southern Medical University, Guangzhou 510280, China; ^2^Department of Pediatrics, The Sixth Affiliated Hospital of Sun Yat-sen University, Guangzhou 510655, China; ^3^Department of Neonatology, Nanfang Hospital, Southern Medical University, Guangzhou 510515, China; ^4^Department of Pediatrics, West China Second University Hospital, Sichuan University, Chengdu 610041, China; ^5^Key Laboratory of Birth Defects and Related Diseases of Women and Children, Ministry of Education, West China Second University Hospital, Sichuan University, Chengdu 610041, China

## Abstract

Uncoupling protein 2 (UCP2) has a cardioprotective role under septic conditions, but the underlying mechanism remains unclear. This study aimed at investigating the effects of UCP2 on the oxidative stress and apoptosis of cardiomyocytes induced by lipopolysaccharide (LPS). First, LPS increased UCP2 expression in cardiomyocytes in a time-dependent manner. LPS increased the production of lactate dehydrogenase (LDH), reactive oxygen species (ROS), and malondialdehyde (MDA) and decreased the level of superoxide dismutase (SOD). However, UCP2 knockdown increased the LPS-induced cardiac injury and oxidative stress. In addition, LPS damaged the mitochondrial ultrastructure and led to the disruption of mitochondrial membrane potential (MMP), as well as the release of mitochondrial cytochrome c. UCP2 knockdown aggravated mitochondrial injury and the release of mitochondrial cytochrome c. LPS increased the protein levels of Bax and cleaved-caspase-3, decreased the protein level of Bcl-2, and upregulated the protein level of mitogen-activated protein kinase. However, upon UCP2 knockdown, the protein levels of Bax and cleaved-caspase-3 increased even further, and the protein level of Bcl-2 was further decreased. The protein level of phosphorylated p38 was also further enhanced. Thus, UCP2 protects against LPS-induced oxidative stress and apoptosis in cardiomyocytes.

## 1. Introduction

Sepsis is the host response to infection and a major cause of mortality worldwide [1–3]. Most deaths in sepsis are attributed to the development of multiple organ failure, and cardiac involvement is very common. Previous studies have indicated that cardiovascular dysfunction is one of the main predictors of morbidity and mortality associated with sepsis [4, 5]. Sepsis patients experiencing myocardial dysfunction have a high mortality of up to 70% [6].

Mitochondrial oxidative stress and apoptosis have been demonstrated to play a critical role in sepsis-induced myocardial dysfunction (SIMD) [7–9]. Generation of reactive oxygen species (ROS) associated with sepsis is known as one of the most deleterious causes of oxidative damage. Three potential sources of ROS have been proposed to be responsible for this release: mitochondrial, xanthine oxidase, and NADPH oxidase [10–12]. In the context of sepsis, ROS generation by the mitochondria further stimulates ROS production in endothelial cells, triggering a vicious cycle of free radical production resulting in a wide variety of reversible and irreversible toxic modifications on biomolecules [12–14]. Excessive production of mitochondrial ROS is responsible for oxidative stress and correlates with the development of SIMD due to the effects on myocardial cells [15–17]. In addition, excessive harmful ROS could trigger mitochondrial oxidative damage and a series of apoptotic events in cardiomyocytes, which eventually lead to cell death [18–20]. Sepsis significantly increases the expression of Bax and Bak in the myocardium, which promotes the release of cytochrome c from the mitochondria [21]. Accumulating evidence strongly suggests that myocardial apoptosis contributes to cardiac dysfunction in sepsis [22, 23]. Thus, agents with antioxidative and antiapoptotic effects may protect cardiomyocytes under septic conditions.

The uncoupling proteins (UCPs) are a family of mitochondrial transport proteins located in the inner mitochondrial membrane, which constitute a vital link between ATP and ROS production [24]. UCP2 and UCP3 are the two important proteins of this family for their protective effects against mitochondrial oxidative damage by reducing the production of ROS. UCP2 is the only ubiquitous isoform, whereas UCP3 is mainly restricted to the skeletal and cardiac muscles. UCP2 not only activated proton leak but also able to export C4 metabolites from mitochondria to the cytosol and regulates substrate oxidation by limiting mitochondrial oxidation of glucose and enhancing glutaminolysis [25, 26]. UCP3 has been suggested to play an important role in regulating fatty acid metabolism and activating proton leak [24, 26]. Although both UCP2 and UCP3 have cardioprotective effects, UCP2 has received more attention in recent years. Increasing studies reported that cardiac UCP2 expression was increased in animal models of sepsis. Endotoxemia in rats and mice induced an increase in cardiac UCP2 mRNA, which is associated with mitochondrial damage, oxidative stress, and cardiac dysfunction [27–29]. Similarly, an increase in cardiac UCP2 mRNA expression, but not UCP2 protein levels, was reported in a rat model of peritoneal sepsis [30]. Additionally, increased myocardial protein expression of UCP2 was observed in a canine and rat model of endotoxin-induced sepsis associated with decreased phosphocreatine/ATP ratios [31–33]. Moreover, recent studies suggested that increased UCP2 expression may actually exert protective effects in cardiomyocytes under septic conditions [34]. Based on previous studies, the downregulation of the UCP2 expression aggravates mitochondrial dysfunction and increases inflammatory response in cardiomyocytes by modulating the activation of mitogen-activated protein kinase (MAPK) pathways [35–37]. Additionally, UCP2 overexpression may reduce apoptotic stress in human umbilical vein endothelial cells and cardiomyocytes by decreasing caspase-3 activity and Bax accumulation [34, 38]. Although these data suggest that UCP2 plays an important role in injured cardiomyocytes, it is unclear whether UCP2 exerts cardioprotective effects on LPS-induced oxidative stress and apoptosis under septic conditions.

To date, little attention has been paid to the impact of UCP2 on the oxidative stress and apoptosis of cardiomyocytes under septic conditions. Therefore, we hypothesized that UCP2 may prevent oxidative stress and apoptosis in cardiomyocytes in a cell model of LPS-induced injury. We used a short hairpin RNA (shRNA) to knock down UCP2 expression in cells to study the effects of UCP2 on cardiac function.

## 2. Materials and Methods

### 2.1. Chemicals and Reagents

Dulbecco's modified Eagle's medium (DMEM) and fetal bovine serum (FBS) were purchased from Gibco (Carlsbad, CA). Trypsin and collagenase were purchased from Sigma-Aldrich (MO, USA). Antibodies against Bax, Bcl-2, cleaved-caspase-3, and phospho-p38 MAPK were purchased from Cell Signaling Technology (MA, USA). Antibodies against cytochrome c was purchased from Abcam (MA, USA). Antibodies against UCP2, Cox-IV, and GAPDH were purchased from Proteintech (IL, USA).

### 2.2. Cell Culture and Adenovirus Transduction

Neonatal rat cardiomyocytes from 1- to 3-day-old Sprague-Dawley rats were isolated and cultured as previously described [39]. The isolated cells were cultured in DMEM supplemented with 10% FBS and 1% penicillin/streptomycin for 3-4 days in a 95% air/5% CO_2_ atmosphere at 37°C. Recombinant adenovirus carrying a shRNA targeting UCP2 (Ad-shRNA-UCP2) and recombinant adenovirus carrying a negative control shRNA (Ad-shRNA-nc) were generated by Obio Technology Company (Shanghai, China). The cardiomyocytes were infected by adenovirus with a multiplicity of infection of 100 according to the manufacturer's instructions. After the adenoviruses were transduced for 72 h, the cells were treated with either phosphate-buffered saline (PBS) or 25 *μ*M LPS for all subsequent experiments and analyses.

### 2.3. Lactate Dehydrogenase (LDH) Assay

LDH, which reflects a loss of membrane integrity of the cardiomyocytes, was detected by a colorimetric assay. Twenty-four hours after cell treatment, approximately 50 *μ*l of culture supernatants was collected, and the LDH activity was measured using commercially available kits (Jiancheng Bioengineering Institute, Nanjing, China) in accordance with the manufacturer's instructions. The absorbance was read at 440 nm on a multifunctional microplate reader.

### 2.4. Measurement of Superoxide Dismutase (SOD) and Malondialdehyde (MDA) Contents

The enzymatic activities of SOD and the MDA content were measured using the corresponding kits (Jiancheng Bioengineering Institute, Nanjing, China). After the cardiomyocytes were subjected to the various treatments, the cells were collected and homogenized in 500 *μ*l PBS using an ultrasonic cell crusher and then centrifuged at 1200 rpm for 10 min at 4°C. The supernatants were collected to determine the SOD and MDA contents with the corresponding kits in accordance with the manufacturer's instructions.

### 2.5. Measurement of Reactive Oxygen Species (ROS) Levels

The intracellular ROS generation in cardiomyocytes was assessed by dihydroethidium (DHE; Invitrogen Molecular Probes). After the cardiomyocytes were subjected to the various treatments, they were loaded with 10 *μ*M DHE according to the manufacturer's instructions at 37°C for 45 min. Finally, cells were examined using a fluorescence microscope (Olympus, Japan), and the intensity of DHE fluorescence was quantified using flow cytometry (BD Biosciences, USA).

### 2.6. Measurement of Mitochondrial Membrane Potential (MMP)

Mitochondrial membrane potential (MMP) in cardiomyocytes was measured using the JC-1 kit (Sigma-Aldrich Co.) according to the manufacturer's instructions. After stimulation, cells were incubated with JC-1 staining solution at 37°C for 25 min and washed three times with JC-1 staining buffer. MMP was assayed with flow cytometry (BD Biosciences, USA). The ratio of red/green JC-1 fluorescence was used as a marker to measure the change in MMP.

### 2.7. Apoptosis Assays

Apoptosis of cardiomyocytes was performed by using an Annexin V-FITC Apoptosis Detection Kit (Dojindo, Shanghai, China) according to the manufacturer's instructions. After the neonatal cardiomyocytes were subjected to the various treatments, they were resuspended in 200 *μ*l Annexin-binding buffer containing 10 *μ*l Annexin V-FITC and 5 *μ*l PI and incubated at 37°C for 15 min in the dark. After incubation, the apoptotic cells were detected using flow cytometry (BD Biosciences, USA).

### 2.8. RNA Analysis

Total RNA from cardiomyocytes was isolated using RNAiso PLUS (TaKaRa Shuzo Co., Kyoto, Japan). The purity of the RNA samples was quantified by a ND1000 spectrophotometer (NanoDrop Inc., Wilmington, DE). cDNAs were synthesized from 0.5 mg of total RNAs using a PrimeScript™ RT reagent Kit (TaKaRa Shuzo Co., Kyoto, Japan). qRT-PCR was performed using SYBR Premix Ex Taq™ (TaKaRa Shuzo Co., Kyoto, Japan) on an ABI 7500 Real-Time PCR System (Life Technologies Corporation, Carlsbad, CA). In addition, each reaction was performed with three technical replicates. The primer sequences are listed as follows: UCP2, forward primer 5′- ACCATTGCACGAGAGGAAGG-3′, reverse primer 5′- TCTTGACCACATCAACGGGG-3′; and glyceraldehyde-3-phosphate dehydrogenase (GAPDH), forward primer 5′-ATCAAGAAGGTGGTGAAGCA-3′, reverse primer 5′-AAGGTGGAAGAATGGGAGTTG-3′.

### 2.9. Western Blot Analysis

Total protein was extracted using a Whole Cell Lysis Assay (KeyGEN, China), and the cytoplasmic and mitochondrial proteins were extracted using a Cytoplasmic and Mitochondrial Protein Extraction kit (Beyotime, China). The protein concentration was determined using a BCA Protein Assay kit (KeyGEN, China). Protein samples (50 *μ*g per lane) were separated using SDS-polyacrylamide gel electrophoresis and transferred to PVDF membranes (Millipore, USA) for 60 min. Subsequently, the membranes were blocked with a solution containing 5% nonfat milk/bovine serum albumin for 90 min at room temperature and then treated with specific primary antibodies at 4°C overnight. Afterwards, the membranes were incubated with a fluorescent secondary antibody for 1 h at room temperature. Then, the membranes were washed three times with TBST and scanned by a LI-COR Odyssey Infrared Imaging System (LI-COR Biosciences, USA). Densitometry was performed using ImageJ software.

### 2.10. Transmission Electron Microscopy

After the cardiomyocytes were subjected to the various treatments, they were collected and fixed in 4% paraformaldehyde and 2% glutaraldehyde in 0.1 M PBS at pH 7.4 overnight at 4°C. The cells were then stained with uranyl acetate and lead citrate and imaged using a Hitachi transmission electron microscope (H-7500; Hitachi, Tokyo, Japan).

### 2.11. Statistical Analysis

SPSS 20.0 was used to analyze the experimental data. Data were collected from three individual experiments and presented as the mean ± SD. Statistical analyses were performed using either one-way analysis of variance (ANOVA) or Student's *t*-test. A *P* value of < 0.05 was considered statistically significant.

## 3. Results

**Figure 1:**
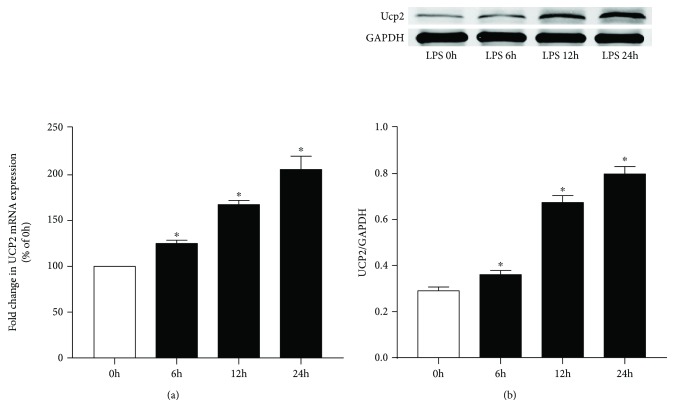
LPS treatment increased UCP2 expression in cardiomyocytes. Cardiomyocytes treated with LPS for varying time periods were analyzed by real-time PCR and western blot analysis. (a) Relative fold change of the mRNA levels of UCP2. (b) Relative fold change of the protein levels of UCP2. Data were collected from three individual experiments and presented as the mean ± SD. ^∗^ *P* < 0.05 vs. the nontreated control.

**Figure 2:**
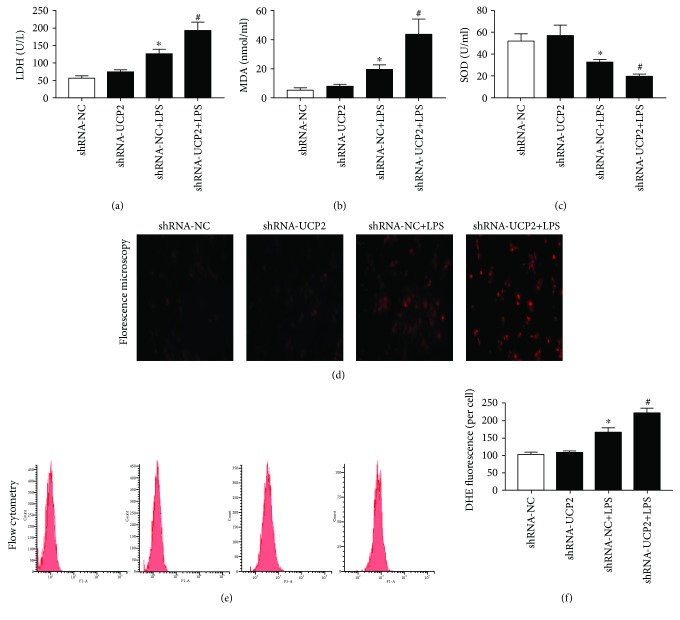
The effect of UCP2 knockdown on LPS-induced cardiomyocyte damage and oxidative stress. Cardiomyocytes were transduced with Ad-shRNA-NC or Ad-shRNA-UCP2 adenovirus followed by treatment with or without 25 *μ*M LPS. (a) Cardiomyocyte damage was determined using the LDH assay. (b, c) The levels of MDA (b) and SOD (c) in the cardiomyocytes were measured using the corresponding kits. (d) ROS production was determined by assessing representative immunofluorescence images. (e, f) Cardiomyocytes were stained with DHE followed by flow cytometry. Data were collected from three individual experiments and presented as the mean ± SD. ^∗^ *P* < 0.05 vs. shRNA-NC, ^#^ *P* < 0.05 vs. shRNA-NC+LPS.

First, we investigated the effects of LPS treatment on UCP2 and UCP3 expression in neonatal rat cardiomyocytes. Cells were incubated with LPS for different periods of time. As shown in Figure 1, UCP2 expression in cardiomyocytes was markedly induced by LPS treatment in a time-dependent manner (*P* < 0.05). As shown in supplementary Figure 1, LPS treatment did upregulate UCP3 expression in cardiomyocytes, but the increasing magnitude was not so remarkable as UCP2. So we focus on the role of UCP2 in cardiomyocytes. The increased levels of UCP2 suggest a potential role for UCP2 in the treatment of cardiomyocytes suffering from LPS insults. We further studied the role of UCP2 in LPS insults. LPS significantly increased the LDH levels (*P* < 0.05) (Figure 2(a)). However, knockdown of UCP2 with shRNA enhanced this increase (*P* < 0.05).

To determine the effects of UCP2 knockdown on cellular redox status in cardiomyocytes, we evaluated the ROS levels, SOD activities, and MDA contents. As shown in Figures 2(b), 2(d), and 2(e), ROS production and MDA content were significantly increased by LPS stimulation (*P* < 0.05). UCP2 knockdown enhanced the increase of ROS and MDA compared with shRNA-NC+LPS (*P* < 0.05). Conversely, the SOD activities were downregulated by LPS, and this downregulation was enhanced by UCP2 knockdown (*P* < 0.05) (Figure 2(c)).

**Figure 3:**
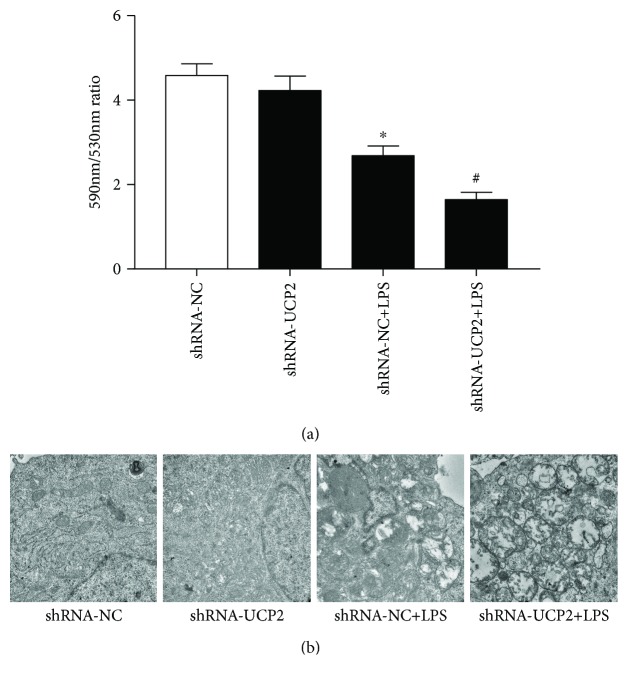
The effect of UCP2 knockdown on mitochondrial function and morphology with LPS treatment. Cardiomyocytes were transduced with Ad-shRNA-NC or Ad-shRNA-UCP2 adenovirus followed by treatment with or without 25 *μ*M LPS. (a) Cardiomyocytes were stained with JC-1 to determine the mitochondrial membrane potential (ΔΨm) by flow cytometry. (b) The mitochondrial ultrastructure was imaged using a transmission electron microscope. Data were collected from three individual experiments and presented as the mean ± SD. ^∗^ *P* < 0.05 vs. shRNA-NC, ^#^ *P* < 0.05 vs. shRNA-NC+LPS.

**Figure 4:**
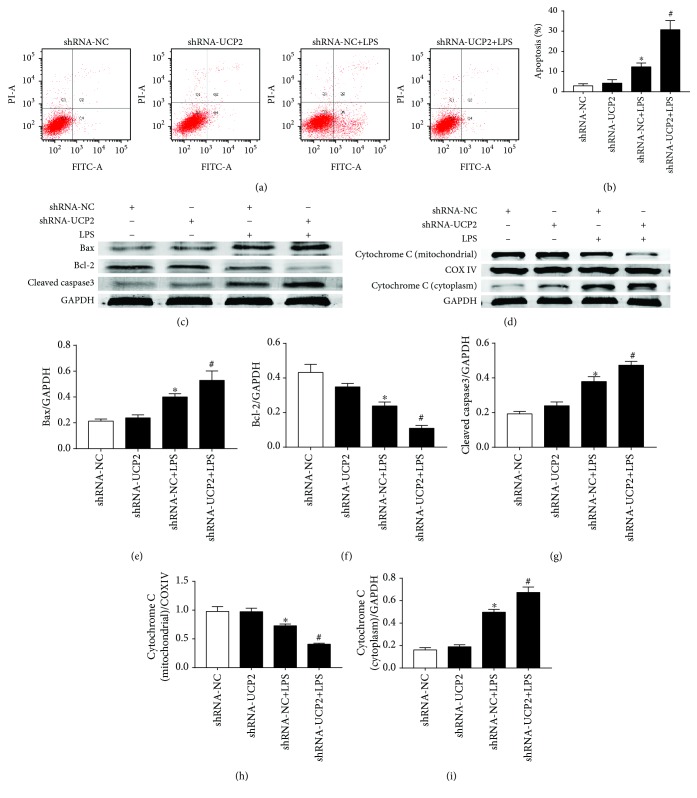
The effect of UCP2 knockdown on LPS-induced cardiomyocyte apoptosis. Cardiomyocytes were transduced with Ad-shRNA-NC or Ad-shRNA-UCP2 adenovirus followed by treatment with or without 25 *μ*M LPS. (a) Cells were stained with Annexin V-FITC and PI followed by flow cytometric analysis. (b) The change in apoptosis rate. (c, d) Effects of UCP2 downregulation on the protein levels of Bax, bcl-2, cleaved-caspase-3 (c), cytochrome c (d), and GAPDH were assessed using western blotting. (e-i) Quantification relative to GAPDH level. Data were collected from three individual experiments and presented as the mean ± SD. ^∗^ *P* < 0.05 vs. shRNA-NC, ^#^ *P* < 0.05 vs. shRNA-NC+LPS.

Disruption of MMP is an early step in cell apoptosis, and the generation of ROS is often accompanied with the loss of MMP [34, 40, 41]. As shown in Figure 3(a), LPS triggered mitochondrial membrane damage and resulted in the loss of MMP (*P* < 0.05). UCP2 knockdown significantly aggravated the loss of MMP (*P* < 0.05). With the MMP collapse, cytochrome c was released from the mitochondria into the cytosol. As shown in Figures 4(d), 4(h), and 4(i), cytochrome c in the mitochondria was significantly decreased, and UCP2 knockdown significantly aggravated this reduction (*P* < 0.05).

As shown in Figure 3(b), ultrastructural analysis of the cardiomyocytes indicated that cells treated with LPS began to show changes in mitochondrial morphology, including swelling and vacuolization of the mitochondria. UCP2 knockdown significantly aggravated these LPS-induced changes.

As shown in Figures 4(a) and 4(b), LPS significantly increased the total cellular rate of apoptosis by 8.1% compared with shRNA-NC (*P* < 0.05). Furthermore, UCP2 knockdown increased the total cellular apoptosis rate by 18.4% compared with shRNA-NC+LPS (*P* < 0.05). Western blot analysis showed that the levels of Bax and cleaved-caspase-3 were significantly increased by LPS (*P* < 0.05) (Figures 4(c), 4(e), and 4(g)). UCP2 knockdown enhanced the increase of these two proteins (*P* < 0.05). Conversely, bcl-2 was downregulated by LPS, and this downregulation was enhanced by UCP2 knockdown (*P* < 0.05) (Figure 4(c)).

**Figure 5:**
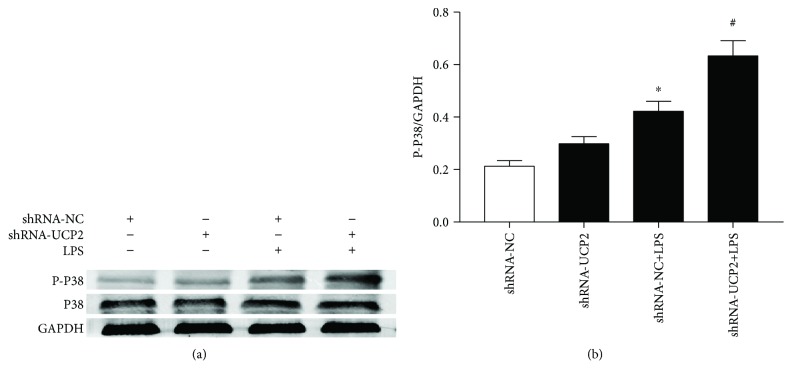
The effect of UCP2 knockdown on LPS-induced activation of MAPK. Cardiomyocytes were transduced with Ad-shRNA-NC or Ad-shRNA-UCP2 adenovirus followed by treatment with or without 25 *μ*M LPS. (a) Effects of UCP2 downregulation on the protein expression of total and phosphorylated p38 were assessed by western blotting. (b) Quantification relative to GAPDH level. Data were collected from three individual experiments and presented as the mean ± SD. ^∗^ *P* < 0.05 vs. shRNA-NC, ^#^ *P* < 0.05 vs. shRNA-NC+LPS.

In addition, we examined the activation of MAPK in cardiomyocytes. As shown in Figure 5, culture with LPS markedly increased the p38 phosphorylation levels, which were further increased in the LPS group infected with Ad-shRNA-UCP2 (*P* < 0.05). The total p38 levels in the Ad-shRNA-UCP2 and Ad-shRNA-NC groups did not differ after normal glucose treatment.

## 4. Discussion

Sepsis-induced myocardial dysfunction (SIMD), a severe complication of sepsis, occurs worldwide and leads to a high rate of mortality [42]. Although many clinical and basic research efforts have been made to understand its pathophysiology for more than 30 years, its pathophysiology is not completely understood. UCP2 and UCP3 belong to a specific mitochondrial inner membrane protein family with the capacity to exert protective actions in the heart by activating proton leak and diminishing ROS emission. Many studies have considered UCP2 and UCP3 to be functionally equivalent [24, 26]. Our present study suggested that UCP2 and UCP3 expression in cardiomyocytes was induced by LPS treatment, but the increasing magnitude of UCP3 was not so remarkable as UCP2. Additionally, a recent study showed that UCP2 plays an important protective role in cardiomyocytes under septic conditions [33, 37]. However, its specific mechanism remains unclear. Thus, the cardioprotective role of UCP2 is worthy of further discussion compared with UCP3. The present study aimed at investigating the effects of UCP2 on the oxidative stress and apoptosis of cardiomyocytes induced by LPS.

Oxidative stress and apoptosis are considered important events of LPS-induced myocardial injury [43, 44]. Although Zheng et al. [37] reported that silencing of UCP2 by siRNA aggravated the damage to mitochondrial morphology and function in cardiomyocytes, the mechanism of UCP2 in LPS-induced myocardial injury has not been fully elucidated. Our present study suggested that knockdown of UCP2 expression substantially increased the LPS-induced oxidative stress and apoptosis in cardiomyocytes, which subsequently influenced cardiomyocyte survival. In addition, UCP2 knockdown significantly aggravated the loss of MMP and the release of mitochondrial cytochrome c. Moreover, UCP2 knockdown suppressed p38 activation in cardiomyocytes. These findings support the hypothesis that UCP2 may protect cardiac cells against LPS-induced oxidative stress and apoptosis in cardiomyocytes through a signaling pathway regulated by the mitochondria.

Oxidative stress plays a major role in the pathogenesis of cardiovascular diseases [45]. Specifically, excessive harmful ROS can trigger mitochondrial oxidative damage in the heart. In this study, UCP2 knockdown enhanced the increases in ROS and MDA, as well as the reduction in cellular SOD levels. These data indicate that UCP2 may reduce ROS production and have antioxidative protective effects on cardiomyocytes in response to LPS. UCP2 may enhance uncoupling function by allowing protons to leak from the intermembrane space into the mitochondrial matrix, which partially relieves the mitochondrial complex dysfunction triggered by LPS stimulation, reduces excessive potential, decreases the interactions between electrons and oxygens, and therefore reduces ROS production [34]. Another possible mechanism is that UCP2 reduces ROS production and limits mitochondrial oxidation of glucose by transporting C4 metabolites out of the mitochondria [25]. Apoptosis is also involved in SIMD, which can be triggered by oxidative stress. There is increasing evidence that the apoptosis in SIMD involves MMP loss, cytochrome c release, and the cleavage of caspase-3, ultimately resulting in cell apoptosis. In the present study, UCP2 knockdown enhanced the upregulation of apoptosis-associated proteins (cleaved-caspase-3 and Bax) and the downregulation of the apoptosis regulator protein bcl-2. Additionally, UCP2 knockdown significantly aggravated the damage to mitochondria and enhanced the release of cytochrome c from the mitochondria. These results indicated that the increased apoptotic stress and mitochondrial injury in cells may be explained by the increase in oxidative stress induced by UCP2 knockdown.

The MAPK signaling pathway is one of the best-understood signaling processes involved in sepsis [46]. p38 MAPK has been identified as a proapoptotic protein that can be activated by inflammation and oxidative stress. Specifically, p38 MAPK is activated by elevated ROS generation in response to LPS. As reported by Yang et al. [47], p38 MAPK activity plays a crucial role in cardiomyocyte apoptosis in SIMD. UCP2 knockdown in cardiomyocytes with a siRNA markedly enhanced the subsequent LPS-stimulated p38 phosphorylation, which promoted oxidative stress and increased apoptotic stress. These data suggest that UCP2 protects cardiomyocytes from LPS-induced cardiac injury by suppressing the activation of p38.

There are some limitations in our study. First, it should be noted that our results were only obtained with cardiomyocytes isolated from neonatal rat. Additionally, the specificity of commercially available antibodies for UCP2 was doubted since UCP2 cannot be detected in adult hearts while some studies found it was expressed [29, 48–51]. In supplemental Figure 3, UCP2 was found highly expressed in the spleen (positive control) and expressed low in the brain (negative control), which suggested that the antibodies for UCP2 had a certain specificity. However, a much better antibody for UCP2 is necessary for further research.

## 5. Conclusion

In conclusion, UCP2 protected cardiomyocytes against LPS-induced oxidative stress and apoptosis by inhibiting p38 MAPK signaling. This protein exhibited antioxidative and antiapoptotic effects through the mitochondrial pathway. These findings provide novel evidence supporting the role of UCP2 and may have important implications for the treatment of SIMD.

## Data Availability

The data used to support the findings of this study are available from the corresponding author upon request.

## Supplementary Materials

Supplementary Materials Figure S1: LPS treatment increased UCP3 expression in cardiomyocytes. Cardiomyocytes treated with LPS for varying time periods were analyzed by real-time PCR and western blot analysis. Data were collected from three individual experiments and presented as the mean ± SD. (a) Relative fold change of the mRNA levels of UCP3. (b) Relative fold change of the protein levels of UCP3. ^∗^ *P* < 0.05 vs. the nontreated control. Figure S2: the efficiency and UCP2 expression of UCP2 knockdown with or without LPS treatment in cardiomyocytes. Cardiomyocytes were transduced with Ad-shRNA-NC or Ad-shRNAUCP2 adenovirus followed by treatment with or without 25 *μ*M LPS. (a) Effects of UCP2 knockdown on the protein expression of UCP2 and GAPDH were assessed by western blotting. (b) Quantification relative to GAPDH level. Data were collected from three individual experiments and presented as the mean ± SD. ^∗^ *P* < 0.05 vs. shRNA-NC, ^#^ *P* < 0.05 vs. shRNA-NC+LPS. Figure S3: the positive control (spleen from rat) and negative control (brain from rat) of UCP2.
