# Supplementary material for: Upregulation of UCP2 Expression Protects against LPS-Induced Oxidative Stress and Apoptosis in Cardiomyocytes
Source: Oxid Med Cell Longev. 2019 Apr 28;2019:2758262. doi: 10.1155/2019/2758262 (PMC6512061; doi:10.1155/2019/2758262)
Supplement: Supplementary Materials — Figure S1: LPS treatment increased UCP3 expression in cardiomyocytes. Cardiomyocytes treated with LPS for varying time periods were analyzed by real-time PCR and western blot analysis. Data were collected from three individual experiments and presented as the mean ± SD. (a) Relative fold change of the mRNA levels of UCP3. (b) Relative fold change of the protein levels of UCP3. ∗ P < 0.05 vs. the nontreated control. Figure S2: the efficiency and UCP2 expression of UCP2 knockdown with or without LPS treatment in cardiomyocytes. Cardiomyocytes were transduced with Ad-shRNA-NC or Ad-shRNAUCP2 adenovirus followed by treatment with or without 25 μM LPS. (a) Effects of UCP2 knockdown on the protein expression of UCP2 and GAPDH were assessed by western blotting. (b) Quantification relative to GAPDH level. Data were collected from three individual experiments and presented as the mean ± SD. ∗ P < 0.05 vs. shRNA-NC, # P < 0.05 vs. shRNA-NC+LPS. Figure S3: the positive control (spleen from rat) and negative control (brain from rat) of UCP2. [file 2758262.f1.pdf]

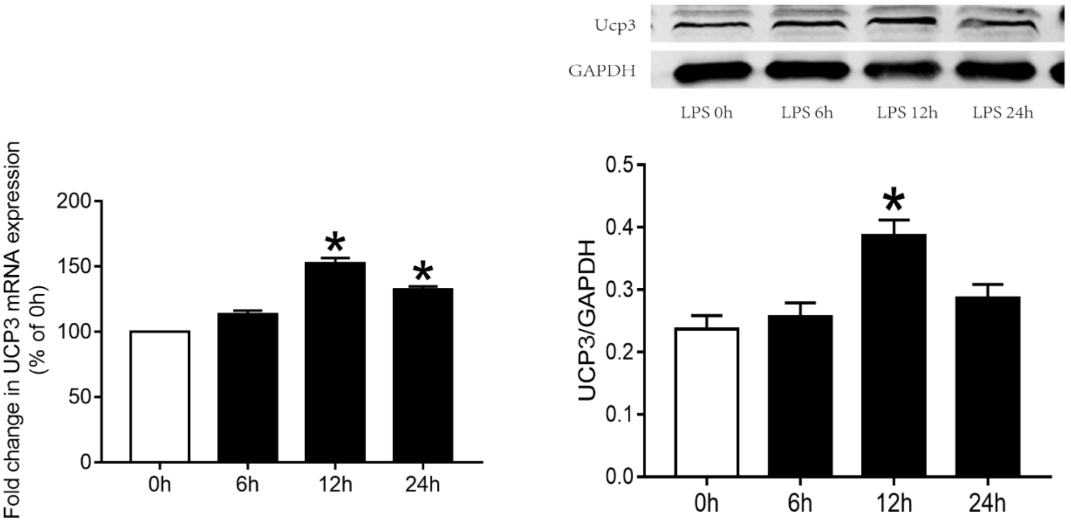


(a) (b)

Supplemental figure. 1: LPS treatment increased UCP3 expression in cardiomyocytes. Cardiomyocytes treated with LPS for varying time periods were analyzed by real-time PCR and western blot analysis. Data were collected from three individual experiments and presented as the mean±SD. (a) Relative fold change of the mRNA levels of UCP3. (b) Relative fold change of the protein levels of UCP3. *P<0.05 vs. the nontreated control.

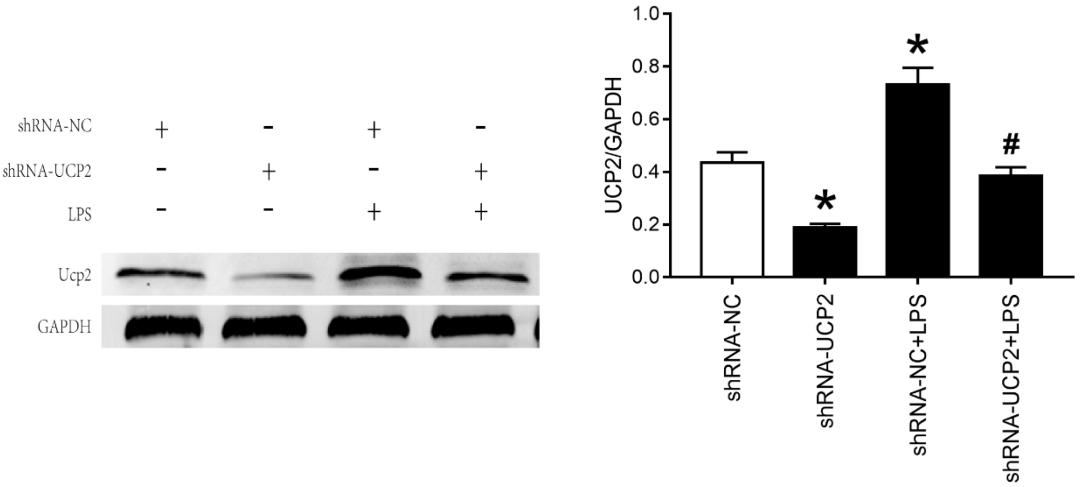


(a) (b)

Supplemental figure. 2: The efficiency and UCP2 expression of UCP2 knockdown with or without LPS treatment in cardiomyocytes. Cardiomyocytes were transduced with Ad-shRNA-NC or Ad-shRNA-UCP2 adenovirus followed by treatment with or without 25 μM LPS. (a) Effects of UCP2 knockdown on the protein expression of UCP2 and GAPDH were assessed by Western blotting. (b) Quantification relative to GAPDH level. Data were collected from three individual experiments and presented as the mean±SD. *P<0.05 vs. shRNA-NC, #P<0.05 vs. shRNA-NC +LPS.

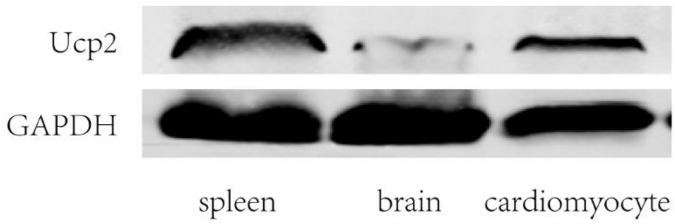


Supplemental figure. 3: The positive control (spleen from rat) and negative control (brain from rat) of UCP2.
